# Correlation between facial morphology and gene polymorphisms in the Uygur youth population

**DOI:** 10.18632/oncotarget.16187

**Published:** 2017-03-14

**Authors:** Huiyu He, Xue Mi, Jiayu Zhang, Qin Zhang, Yuan Yao, Xu Zhang, Feng Xiao, Chunping Zhao, Shutao Zheng

**Affiliations:** ^1^ Department of Prosthodontics, The First Affiliated Hospital of Xinjiang Medical University, Urumqi 830054, Xinjiang Uygur Autonomous Region, People's Republic of China; ^2^ Department of Stomatology, The First People's Hospital of Kashi, Kashi 844000, Xinjiang Uygur Autonomous Region, People's Republic of China; ^3^ Clinical Medical Research Institute, The First Affiliated Hospital of Xinjiang Medical University, Urumqi 830054, Xinjiang Uygur Autonomous Region, People's Republic of China

**Keywords:** three-dimensional grating facial scanning technology, facial morphology, gene polymorphism, SNaPshot technology

## Abstract

Human facial morphology varies considerably among individuals and can be influenced by gene polymorphisms. We explored the effects of single nucleotide polymorphisms (SNPs) on facial features in the Uygur youth population of the Kashi area in Xinjiang, China. Saliva samples were collected from 578 volunteers, and 10 SNPs previously associated with variations in facial physiognomy were genotyped. In parallel, 3D images of the subjects’ faces were obtained using grating facial scanning technology. After delimitation of 15 salient landmarks, the correlation between SNPs and the distances between facial landmark pairs was assessed. Analysis of variance revealed that *ENPP1* rs7754561 polymorphism was significantly associated with RAla-RLipCn and RLipCn-Sbn linear distances (*p* = 0.044 and *p* = 0.012, respectively) as well as RLipCn-Stm curve distance (*p* = 0.042). The *GHR* rs6180 polymorphism correlated with RLipCn-Stm linear distance (*p* = 0.04), while the *GHR* rs6184 polymorphism correlated with RLipCn-ULipP curve distance (*p* = 0.047). The *FGFR1* rs4647905 polymorphism was associated with LLipCn-Nsn linear distance (*p* = 0.042). These results reveal that *ENPP1* and *FGFR1* influence lower anterior face height, the distance from the upper lip to the nasal floor, and lip shape. *FGFR1* also influences the lower anterior face height, while *GHR* is associated with the length and width of the lip.

## INTRODUCTION

Facial features and expressions are key elements of non-verbal communication and mutual identification, and show significant particularities and differences between individuals. Facial morphological characteristics are related to the expression of several genes [1–5], and closely linked to the activity of several signaling pathways, including BMP, SHH, FGF, ENPP1 and Wnt/β-catenin [6–9]. Notwithstanding, our current understanding of the development of facial morphology comes mainly from anatomical research, and the possible influence of single base DNA mutations (single-nucleotide polymorphisms, SNPs) remains scantily explored. In recent years, however, the possible association between gene polymorphisms and facial morphology, related in particular to differences in facial features among ethnic groups, has become an attractive research topic.

A few studies have addressed the genetic regulation of facial morphogenesis and put forward a series of candidate genes. *IRF6* (interferon regulatory factor 6) is a key factor in the growth and development of keratinocytes. Peng et al. [10] found that there was a significant correlation between the expression of *IRF6* and the morphology of the mouth in Chinese Han women. *ENPP1* (ectonucleotide pyrophosphatase/phosphodiesterase 1) encodes an enzyme that negatively regulates bone mineralization. When mutated at 5′UTR and 3′UTR, it leads to a change in the height of the upper face [11]. The *GHR* (growth hormone receptor) gene affects the normal growth and development of the human body. Two GHR genetic variants, Pro561Thr (rs6184) and I526L (rs6180), have been shown to alter mandible height in an East Asian population [11]. *FGFR1* (fibroblast growth factor receptor 1) affects normal facial morphology development in humans, and is associated with the cephalic index in multiple populations [11].

The Kashi area is located in the southwest of the Xinjiang Uygur Autonomous Region in northwest China, where the Uygur people are the largest ethnic minority group. The Uygur population presents a Mongolian and Caucasian mixed ancestry, and their facial features, especially the orbital contours resembling Caucasian and European ethnic populations, contribute to the characteristic Uygur facial physiognomy. Guo et al. [12] analyzed high-resolution 3D images of soft-tissue facial forms in four Eurasian populations, including Han Chinese, Tibetans, Uygur and Europeans. Their results showed that among-population differentiation was higher for soft-tissue facial forms than for genome-wide genetic loci. High-resolution data analysis showed that Europeans and Han people had significant differences in the nose, eyebrows region, and cheeks. Thus, we used a high-resolution 3D image acquisition system to study the effect of SNPs on facial morphology in a sample of the Uygur population.

## RESULTS

Hardy-Weinberg equilibrium analysis. Hardy-Weinberg equilibrium (HWE) tests were performed using the exact test to assess the representativity of the population. Through SNP analyses, we found that *ENPP1* rs7773292 did not meet the HW equilibrium, while all other mutation sites did. Linkage disequilibrium (LD) analysis was done in 578 individuals. For the *ENPP1* gene, *ENPP1* rs7773292 was cut off at 5% HWE p level, so 3 SNP loci were included in the LD analysis, namely rs6925433, rs6569759, and rs7754561. The D’ values of rs6925433 and rs6569759, rs6569759 and rs7754561, and rs6925433 and rs7754561 were 0.14, 0.14, and 0.07, respectively, denoting a low degree of linkage disequilibrium. For the *GHR* gene, the D’ value of rs6180 and rs6184 was close to 1, denoting a high level of LD between the sites. Thus, rs6180 and rs6184 sites may be closely linked. For the *FGFR1* gene, the D’ value of rs4647905 and rs3213849 was 0.67 (low LD). For the *IRF6* gene, the D’ value of rs642961 and rs2236907 was 0.93 (high LD).

### Correlation between SNPs and facial landmark distances

The distance between pairs of facial landmarks on 3D images was measured and contrasted with matched SNP genotyping data from study subjects.

### ENPP1 SNPs

Through analysis of variance, we found a significant association between the *ENPP1* rs7754561 polymorphism and the linear distance of both RAla-RLipCn (*p* = 0.044) and RLipCn-Sbn (*p* = 0.012). This SNP also influenced the curve distance of RLipCn-Stm (*p* = 0.042) (Table 1). The linear distance of RAla-RLipCn was longer (31.14 ± 2.86 mm) in individuals carrying the GA genotype than in those with the GG (30.88 ± 2.44 mm) or AA (30.13 ± 1.99 mm) genotypes. The linear distance of RLipCn-Sbn was also longer for the GA genotype (37.80 ± 3.06 mm), compared with GG (36.77 ± 3.49 mm) and AA (36.61 ± 3.16 mm) genotypes. A similar trend was observed the curve distance of RLipCn-Stm, where the GA genotype was associated with a longer inter-landmark distance (27.68 ± 5.13 mm) than that measured for the GG (26.30 ± 2.87 mm) and AA (26.73 ± 2.86 mm) genotypes (Table 1). In all cases, significant differences were detected between the GA and the AA genotypes.

**Table 1:** Association between rs7754561 in the ENPP1 gene and facial landmark pairs distances

SNP rs7754561	Landmark distance (mm)		
	RAla-RLipCn(s)	RLipCn-Sbn(s)	RLipCn-Stm(c)
G/G	30.88 ± 2.44	36.77 ± 3.49	26.30 ± 2.87
G/A	31.14 ± 2.86	37.80 ± 3.06	27.68 ± 5.13
A/A	30.13 ± 1.99	36.61 ± 3.16	26.73 ± 2.86
*F*	3.14	4.47	3.20
*P*	0.044	0.012	0.042

s: straight-line distance; c: curve distance.

*p* < 0.05 indicates statistical significance.

### FGFR1 SNPs

An association was found between the *FGFR1* rs4647905 polymorphism and the linear distance of LLipCn-Nsn (*p* = 0.042; Table 2). Distances for this landmark pair were 78.02 ± 6.36 mm for the GG genotype, 79.59 ± 5.08 mm for the CG genotype, and 77.77 ± 6.01 mm for the CC genotype; the mean inter-landmark distance of individuals carrying the CG genotype was significantly longer than for those carrying the CC genotype.

**Table 2:** Association between rs4647905 in the FGFR1 gene and facial landmark pairs distances

SNP rs4647905	Landmark distance
	LLipCn-Nsn(s)
G/G	78.02 ± 6.36
C/G	79.59 ± 5.08
C/C	77.77 ± 6.01
F	3.21
P	0.042

s: straight-line distance.

*p* < 0.05 indicates statistical significance.

### GHR SNPs

A significant association was detected between the *GHR* rs6180 polymorphism and the linear distance of RLipCn-Stm (*p* = 0.04; Table 3). Distances were 28.34 ± 7.2 mm for the AA genotype, 26.98 ± 3.2 for the CA genotype, and 26.59 ± 3.1 mm for the CC genotype, and the mean distance in the AA genotype was significantly longer than that of the CC genotype. *GHR* rs6180 was also associated with the curve distance of RLipCn-Stm, i.e. 27.27 ± 7.07 mm for the AA genotype, 25.88 ± 2.95 mm for the CA genotype, and 25.64 ± 2.92 mm for the CC genotype; the mean distance for the AA genotype was significantly longer than that of the CC genotype (Table 3).

**Table 3:** Association between rs6180 and rs6184 in the GHR gene and facial landmark pairs distances

SNP rs6180	Landmark distance		SNP rs6184	Landmark distance
	RLipCn-Stm(s)	RLipCn-Stm(c)		RLipCn-ULipP(c)
A/A	28.34 ± 7.2	27.27 ± 7.07	C/C	31.01 ± 3.22
C/A	26.98 ± 3.2	25.88 ± 2.95	C/A	32.27 ± 2.88
C/C	26.59 ± 3.1	25.64 ± 2.92	A/A	28.76
F	3.25	3.25	F	3.09
P	0.04	0.04	p	0.047

s: straight-line distance; c: curve distance.

*p* < 0.05 indicates statistical significance.

Lastly, a significant association was found between the *GHR* rs6184 polymorphism and the curve distance of RLipCn-ULipP (*p* = 0.047). Mean curve distance was 31.01 ± 3.22 mm in the CC genotype, 32.27 ± 72.88 mm in the CA genotype, and 28.76 mm in the AA genotype.

## DISCUSSION

Facial morphology is one of the most easily recognizable features of human beings. While the main genetic pathways defining craniofacial morphogenesis have been elucidated, the extent to which small genetic variations influence facial physiognomy is largely unknown. This study collected SNP genotyping data and 3D facial images from 578 Uygur volunteers (220 males and 358 females, 18 to 25 years old) from the Kashi area of Xinjiang, China, to analyze the relationship between gene polymorphisms and facial morphology. Our results showed that rs7754561 in the *ENPP1* gene, rs4647905 in the *FGFR1* gene, and rs6180 and rs6184 in the *GHR* gene are associated with morphometric facial features.

### ENPP1 polymorphisms

Ectonucleotide pyrophosphatase/phosphodiesterase 1 (*ENPP1*), a membrane-bound ectoenzyme, is one of the key enzymes controlling bone mineralization through its modulation of Pi/PPi levels. The activity of this enzyme leads to production of PPi by catalyzing the hydrolysis of the phosphodiester I bond of nucleoside triphosphates [13]. *ENPP1* expression correlates with osteoblast differentiation, and osteoblast cultures overexpressing *ENPP1* contain elevated amounts of PPi and show reduced mineral formation [14]. Ermakov et al. [11] investigated the association of polymorphisms in the *ENPP1* locus with normal variability of craniofacial phenotypes in 1,042 Western Eurasian individuals, and found that *ENPP1* gene polymorphisms were associated with upper facial height. Additionally, associations were detected between head breadth and lower face height, and markers residing in or close to the promoter and 3′UTR of the *ENPP1* gene.

In our research, there was a significant difference in the linear distance between RAla-RLipCn for the *ENPP1* rs7754561 polymorphism. When the AA, instead of the GA, genotype was present, the linear distance between the nose and the mouth became shorter. As the distance between the sides of the middle nose to the lips is shortened, the distance from the nose to the lips is also shortened, thus locally influencing lower anterior face height. We found also a significant association between rs7754561 and the linear distance of RLipCn-Sbn. Individuals with GG or AA, rather than GA genotypes, showed a shorter linear distance from the nasal floor to the right mouth corner; in the lower anterior face, the distance from the nasal floor to the upper lip is shortened, making the philtrum shorter, increasing the upper lip height, and shortening the width of the lips. The *ENPP1* rs7754561 polymorphism was also correlated with the curve distance of RLipCn-Stm when the genotype was GA, making the distance from the right lip corner to the stomion longer. Consequently, the width between the two lips’ corners, and the width of the stomion, becomes longer. Thus, the ENPP1 rs7754561 polymorphism was associated with variations in the lower anterior face landmarks such as the distance of the subnasale point to the chin, the distance from the subnasale to the lips, and the width of the lips. Based on these observations, we hypothesize that the putative functional genetic variant in this region, most likely in the 3′UTR, affects the stability of *ENPP1* mRNA, which in turn influences the local Pi/PPi levels and bone mineralization. This likely cause generalized effects on skeletal development, affecting facial morphological features.

### FGFR1 polymorphisms

Fibroblast growth factors play important roles in the differential growth of the skull, brain, and facial prominences. Gómezvaldés et al. [15] evaluated the relationships between *FGFR1* polymorphisms and cephalometric measurements and indices in one Mexican Native and two mestizo Mexican populations, and found a tendency for a decrease in cephalic index in individuals homozygous for the allele rs4647905C; when General Linear Model analyses were performed, a statistically significant association was found between four SNPs in *FGFR1* and head length in the mestizo population. In our research, we found that there was a significant association between the *FGFR1* rs4647905 polymorphism and the linear distance of LLipCn-Nsn, affecting the middle anterior face height, and the distance of the upper lip to the subnasale. We surmised that when mutated, this allele shortens the distance from the right lip corner to the nasion. Thus, the middle anterior face height will be reduced, affecting the height of the nasal dorsum and the distance between the upper lip and the nasal floor.

### GHR polymorphisms

Bayram et al. [16] evaluated allelic and genotype frequencies of the P561T and C422F polymorphic sites of the *GHR* gene and their association with mandibular prognathism, and found that effective mandibular length (condylion-gnathion) and lower face height (anterior nasal spina-menton) were associated with the P561T variant. Zhou et al. [17], on the other hand, used quantitative trait locus mapping methods to evaluate the relationship between craniofacial morphology and SNPs in *GHR* in an unselected, healthy Chinese population. Their results showed that individuals with the genotype CC of polymorphism I526L had a significantly greater mandibular ramus length (condylion-gonion/articulare-gonion) than those with genotypes AC or AA. Our results indicated that rs6180 in the *GHR* gene influences the width of the oral fissure. *GHR* rs6184, in contrast, was associated with the height and width of the upper lip, and also influenced the protrusion of the lip.

### 3D facial imaging and morphometry

Three-dimensional grating facial scanning technology is a new non-contact scanning method, by which point cloud data can be converted into three-dimensional images that can be directly processed by specialized image analysis software. Optical scanning technology has many advantages, as it allows non-invasive, highly accurate and fast data acquisition, and is therefore considered a very promising facial image acquisition and measurement technology [18, 19]. Three-dimensional grating facial scanning technology is currently the preferred method for facial morphometric analysis.

In conclusion, this is the first study to assess the effect of gene polymorphisms on facial morphology in the Uygur population from the Kashi area of Xinjiang, China. The results revealed that *ENPP1* and *FGFR1* gene polymorphisms are associated with the lower anterior face height, the distance between the upper lip and nasal floor, and lip shape. *FGFR1* SNPs may also influence the lower anterior face height, while *GHR* gene polymorphisms are associated with the length and width of the lips.

## MATERIALS AND METHODS

### Ethics statement

Sample collection in this study was carried out with the approval of the ethics committee of the First Affiliated Hospital of Xinjiang Medical University and in accordance with the standards of the Declaration of Helsinki. Written informed consent was obtained from every participant.

### Sample collection

807 volunteers aged 18–25 years old from the Uygur population in the Xinjiang Kashi area participated in the study. We removed 177 individuals based on the exclusion criteria. 37 individuals were further excluded due to poor DNA sample quality. In addition, 15 subjects with a typing call rate <95% were excluded. Thus, 578 individuals (358 females, 220 males) were available for 3D imaging and successful genotyping of the 10 candidate SNPs. 3 ml of saliva was collected from each participant for DNA extraction.

Sample inclusion criteria included: 1) no dentition defects; 2) absence of dental caries caused by a dentition defect and altered jaw gum height; 3) no facial outgrowths or deformities. Exclusion criteria included: 1) Individuals with obvious health problems or any history of facial surgery; 2) Refined facial images after obvious distortions did not allow reconstruction of actual facial features.

### DNA extraction and genotyping

Based on a new 3D face reconstruction method utilized by Peng et al., that enables subtle differences to be detected at high resolution in 3D images, we analyzed the associations of 10 candidate SNPs with common facial morphological variations [10]. The SNP analyzed were: *ENPP1* gene, rs7773292, rs6925433, rs6569759, and rs7754561; *GHR* gene, rs6180 and rs6184; *FGFRl* gene, rs4647905 and rs3213849; *IRF6* gene, rs642961 and rs2236907 (Table 4).

**Table 4:** Basic characteristics of selected variants

SNPs	Gene	Allele	Chr	Position	HWE-p	Reference
rs7773292	ENPP1	C/T	6	132099761	0.0439	[10]
rs6925433	ENPP1	A/G	6	132119366	0.9647	[10]
rs6569759	ENPP1	A/G	6	132133116	0.6407	[10]
rs7754561	ENPP1	A/G	6	132212694	1	[10]
rs6180	GHR	A/C	5	42719239	0.3297	[10]
rs6184	GHR	A/C	5	42719344	1	[10]
rs4647905	FGFR1	C/G	8	38272542	0.6102	[10]
rs3213849	FGFR1	A/G	8	38326046	0.7746	[10]
rs642961	IRF6	A/G	1	209989270	0.1457	[10]
rs2236907	IRF6	A/C	1	209971628	0.5128	[10]

Genomic DNA was extracted from saliva following a modified SDS-based DNA extraction method. DNA concentration was measured with a NanoDrop spectrophotometer. Primers were designed using the web-based Primer3 software (Table 5). SNP genotyping was performed with the SNaPshot multiplex system on an ABI3130xl genetic analyzer (Applied Biosystems).

**Table 5:** Primer sequences of selected variants

SNP	Primer Sequence
rs2236907F	CAACCCCCTACGGGAGATTTCA
rs2236907R	GGCTCTGGTTCTGGGTTGGTCT
rs3213849F	CAATGCGCTACGAGGGGTCTC
rs3213849R	CGCGGCTGGGGAACTACAAG
rs4647905F	CACCCATGCAACTAGCCGACTT
rs4647905R	GTAAGGGCTGGCCTGGTTTGAG
rs6180_rs6184F	AATGTGACATGCACCCGGAAAT
rs6180_rs6184R	GAGGCCCTGTGGGGACTGTACT
rs642961F	GTCATGAAGGGGAACCTGAGGA
rs642961R	TGCTCTGAGCCTGAGCGAAACT
rs6569759F	GAGGGAATGAGATGGGCAGAAGTA
rs6569759R	CGCTGCTCTGTCCCCTTATTTTT
rs6925433F	ATTGAGGATCCAGCCCCTTCTT
rs6925433R	GGTGTACCAGTTATGTCTGCAAAGGAT
rs7754561F	ACATGCCACCAATCACCTCACA
rs7754561R	AAGTCTTTGCAGCTGGCCCTTA
rs7773292F	CCCTTCCCCTTCATCCCTCTCT
rs7773292R	GGCACAGAAGCTGATGGCATTT

### High density 3D facial image collection and registration

Facial 3D data was acquired using Geomagic's non-contact facial three-dimensional raster scanner (3D Systems, SC, USA). The subjects were in a sitting position and the scanning distance was 1.2 m. Both men and women were instructed to have their foreheads exposed, a relaxed and neutral facial expression, and to look at the top of the scanner's three-point objective lens. Face scanning precision was set to 0.02 mm, capturing 50,000 points per second. Upon completion of the scan, the subject's face CAD model was exported in STL format.

### Data processing of 3D scanned images

**Figure 1:**
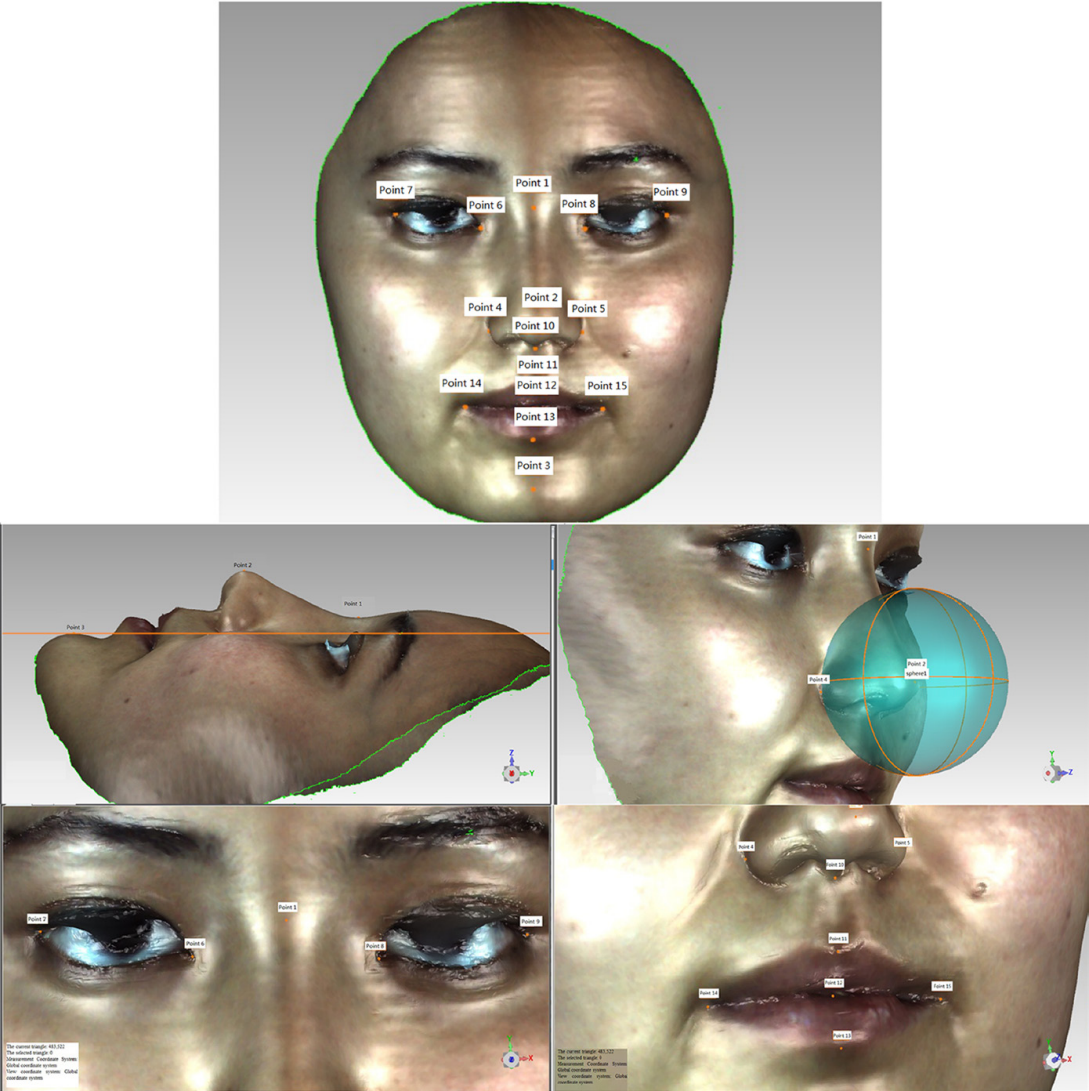
Facial landmarks extracted from 3D imaging The 15 salient facial landmarks defined in 3D images were: 1, Nasion point (Nsn); 2, Pronasale (Prn); 3, Chin point (ChiP); 4, Left Alare (LAla); 5, Right Alare (RAla); 6, Left internal canthus (LIntCan); 7, Left external canthus (LExtCan); 8, Right internal canthus (RIntCan); 9, Right external canthus (RExtCan); 10, Subnasale (Sbn); 11, Upper lip point (ULipP) 12, Stomion (Stm); 13, Lower lip point (LLipP); 14, Left lip corner (LLipCn); 15, Right lip corner (RLipCn).

**Figure 2:**
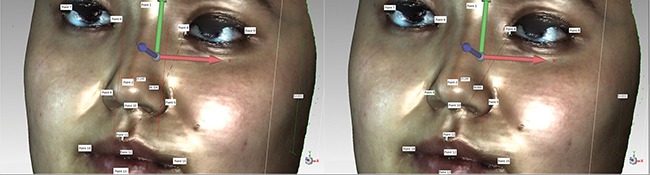
Distance measurements of 3D facial image landmarks

3D facial scanned images were imported into Geomagic studio software. Images were refined, missing parts of the scan were filled, and image outlines were defined to create accurate point objects. The software automatically recognizes 15 salient facial landmarks: Pronasale (Prn); Nasion point (Nsn); Subnasale (Sbn); Chin point (ChiP); Left external canthus (LExtCan); Left internal canthus (LIntCan); Right internal canthus (RIntCan); Right external canthus (RExtCan); Left Alare (LAla); Right Alare (RAla); Right lip corner (RLipCn); Left lip corner (LLipCn); Stomion (Stm); Upper lip point (ULipP); and Lower lip point (LLipP) (Figure 1). Geomagic studio analysis tool was used to measure the distance between two landmarks (Figure 2), namely RAla-RLipCn, RLipCn-ULipP, RLipCn-Sbn, LAla-LLipP, ULipP-LLipP, LLipCn-Sbn, RLipCn-Prn, RLipCn-LLipP, RLipCn-Sbn, RLipCn-Chip, Chip-LAla, RLipCn-Nsn, LLipCn-Nsn, ULipP-RIntCan, ULipP-RExtCan, and RLipCn-Stm. Straight-line (linear) and curve distances were measured separately.

### Statistical analysis

Hardy-Weinberg equilibrium tests were performed using the exact test to assess the genetic variability of the population. *p* = 0.05 was set as the significance level threshold for the candidate gene strategy; *p* > 0.05 indicated that the population surveyed reached genetic balance.

All the data were analyzed by SPSS19.0 statistical software. Measurement data is represented by $\bar{X}$± *SD*. Comparison between groups was performed using analysis of variance, and two-pair comparisons were carried out using the LSD *t* test method. A *p-value* < 0.05 was considered statistically significant.
